# On Cutting off the Tails of Leeches

**Published:** 1816-05

**Authors:** 


*For the London Medical and Physical Journal.*

### *On Cutting off the Tails of Leeches;* by S. M.

THE practice of cutting off the tails of leeches, to promote an increased flow of blood, is by no means novel. It has been familiar to me since I was a very tyro in medical studies, from the perusal of "Heister's Surgery," which was first published in 1739: he says, "if it be necessary to draw a large quantity of blood, you must ........ cut off the tails of those which are drawing with a pair of scissars, by which means the blood will run through them, and they will draw almost as long as you please."

But this practice is to be traced much farther back. Tiberio Malsi, who published at Naples, in 1629, in folio, his "Nuova Prattica della Decoratoria Manuale, et della Sagnia; l'una a Barbieri, et l'altra a Chirurgici singolarmente necessaria, &c." mentions the same mode of managing leeches, and speaks of it as an old custom, for he says, "the *ancients* used a kind of pincers for this purpose, which to me does not appear very proper, the same thing being effected more easily by a pair of scissars." The whole paragraph runs thus:—"Ma perche questo modo di far' uscire il sangue, reca al patiente tal'hora molto travaglio per lo lungo tempo, che occorre stare sedente a cotal guisa; e tal volta anco si ritrovano le farze de gl'infermi assai deboli, et diminute, perciò hò stimato qùi altri modi annotare, per li quali senza tanto travaglio, l'uscita libera del sangue havor si possa. Et il primo si è, che stando le sanguesughe succhiando, lor si dia una forficata per lungo nell'estremo della coda, se bene con destrezza, acciò nel succhiar loro il sangue goccioli in un vasetto a ciò pressarato. Gli Antichi per quest'effetto si valevano d'una certa tenagliuola; il che a me non pare motto a proposito, potendosi haver l'intento a più facilmente, con le forbici."—Lib. terzo. p. 146.

*Half-Moon-street;*

